# The histone genes cluster in *Rhynchosciara americana* and its transcription profile in salivary glands during larval development

**DOI:** 10.1590/1678-4685-GMB-2015-0306

**Published:** 2016-10-10

**Authors:** Fábio Siviero, Paula Rezende-Teixeira, Alexandre de Andrade, Roberto Vicente Santelli, Glaucia Maria Machado-Santelli

**Affiliations:** 1 Departamento de Biologia Celular e Desenvolvimento, Instituto de Ciências Biomédicas, Universidade de São Paulo, São Paulo, SP, Brazil.; 2 Departamento de Bioquímica, Instituto de Química, Universidade de São Paulo, São Paulo, SP, Brazil.

**Keywords:** Rhynchosciara, histone cluster, Sciaridae, codon usage

## Abstract

In this work we report the characterization of the *Rhynchosciara americana* histone genes cluster nucleotide sequence. It spans 5,131 bp and contains the four core histones and the linker histone H1. Putative control elements were detected. We also determined the copy number of the tandem repeat unit through quantitative PCR, as well as the unequivocal chromosome location of this unique locus in chromosome A band 13. The data were compared with histone clusters from the genus *Drosophila*, which are the closest known homologues.

## Introduction

*Rhynchosciara americana* is a dipteran belonging to the family Sciaridae, commonly known as dark-winged fungus gnats. These are known for its exuberant polytene chromosomes and developmentally regulated DNA amplification *loci* present in several tissues, the so-called DNA puffs. *R. americana* has been the object of research since the 1950s (Nonato and Pavan, 1951), but besides the well-known physiology of its polytene chromosomes and their puffs (Santelli *et al*., 1991, 2004; Frydman *et al*., 1993, Penalva *et al*., 1997), the underlying overall molecular biology and evolution are poorly understood.

In *R. americana* salivary glands, the last cycle of DNA replication encompasses a gene amplification phenomenon generating DNA puffs (Breuer and Pavan, 1955; Ficq and Pavan, 1957; Rudkin and Corlette, 1957). This well-documented polyteny cycle lasts for 5 or 6 days. (Machado-Santelli and Basile, 1973, 1975). Due to its long duration, it is possible to precisely follow the replication and transcription processes. However, several structural characteristics of these unusual chromosomes present in Sciaridae are still unknown, such as the amplification control elements.

We started to focus on the histone class of genes as an approach to better understand chromatin structure aspects in the salivary glands of *Rhynchosciara*, and to provide molecular markers for this species. The replication-dependent histones and their variants are common chromatin components of great interest because of their involvement in the modulation of the chromatin transcriptional status and with the replication process.

Data from several laboratories have implicated regulatory regions of the histone genes as targets for factors acting under the control of the Cdk2/cyclin E complex (Ewen, 2000). In mammals, the factor NPAT, identified as substrate of that complex, is a linker element between cell cycle regulators and histone gene transcriptional activation (Zhao *et al*., 2000; Gao *et al*., 2003). Identification of Cajal bodies (CB) participating in this process, as well as the Stem Looping Binding Protein (SLBP), brought new elements to the understanding of the cell cycle-dependent activation of the transcriptional program (G1–S transition) (Marzluff, 2005).

Histones, as the main chromatin organizing proteins, are extremely conserved among eukaryotes. Due to their slow rate of modification through evolution they became landmarks for phylogenetic analysis of distant organisms (Thatcher and Gorovsky, 1994). Usually, histone genes are arranged in two forms: (1) in a cluster containing the five histone genes repeated in tandem several times, such as in *Drosophila* and *Chironomus*, or (2) dispersed in the genome forming incomplete clusters or as single, isolated genes, like in humans (Matsuo and Yamazaki, 1989; Nagoda *et al*., 2005; Hankeln and Schmidt, 1991).

Previous studies have identified the *R. americana* histone gene cluster using a *D. hydei* probe, and semi-quantitative Southern blotting experiments were able to estimate its copy number as approximately 150 tandemly arranged units. Fluorescent *in situ* hybridization analysis localized its chromosomal site in region 13 of chromosome A, near the pericentromeric region (Santelli *et al*., 1996).

To advance the study of *R. americana* histone genes, we now report the complete sequence of the *R. americana* histone gene cluster and the description of its canonical and putative regulatory elements. The data are also compared with counterparts in the genus *Drosophila* genus, due the phylogenetic proximity between the two genera.

## Material and Methods

### Library construction

A recombinant phage λDASHII (Stratagene) – RaHis (Santelli *et al*., 1996) previously isolated using a *Drosophila hydei* histone probe was cut with *Not*I restriction enzyme and the isolated insert was extracted with phenol. Approximately 50 μg of this DNA was sheared using the nebulization method (shotgun strategy) and fragments were separated in low melting point agarose and size selected (1.0 - 1.5 kb). Approximately 2 μg of DNA were then treated with Klenow/PNK enzyme (Amersham/Pharmacia) in a blunting reaction and cloned in pUC18 (Sigma) using T4 DNA Ligase (Gibco).

### Sequencing and assembly

Recombinant *E. coli* DH5α selected by IPTG/XGAL grown in 1 mL of Circle Grow (USB) medium were pelleted (2,200 x g for 10 min) in microplates, following SDS/NaOH (10%/0.2 M) lysis and potassium acetate (3 M) debris precipitation. The lysate was filtered using a MAGV N22 filter plate (Millipore). The plasmid solution was precipitated with isopropanol, washed with ethanol 80% and finally suspended in 30 μL of 10 mM Tris. Sequencing reactions were performed in a PCR GeneAmp 9700 thermocycler (Perkin Elmer), using BigDye terminator technology (96 °C for 1 min, 35 cycles of 96 °C for 20s, 50 °C for 1min and- 60 °C for 4 min). The samples were precipitated with ethanol/Na acetate (3M) supplemented with glycogen (1g/L) and run in an ABI 377 automatic sequencer (Applied Biosystems).

The electropherograms were analyzed and assembled using Phred, Phrap and Consed software packages (Ewing *et al*., 1998; Ewing and Green, 1998; Gordon *et al*., 1998). We adopted the standard Phrap assembly parameters and considered a sequence as of high quality if it had at least 150 contiguous bp with quality ≥ 20 inferred by Phred. The error estimated by the Phred/Phrap/Consed suite for this assembly was 1/10,000 bp.

### Sequence analysis

The initial analyses were carried out through Genscan software (Burge and Karlin, 1997), posterior sequence comparisons, dot-maps and pattern searches were performed with Emboss packages (Rice *et al*., 2000). ClustalX was utilized to build alignments (Thompson *et al*., 1997).

Promoter prediction was made with the Neural Network Promoter Prediction service (http://www.fruitfly.org/seq_tools/promoter.html) (Reese, 2001). CodonW software (Peden, 1999) (http://codonw.sourceforge.net/) was used to generate codon usage tables.

The sequences of histone gene clusters from *Drosophila hydei* (X17072), *Drosophila melanogaster* (X14215) and *Chironomus thummi* (X56335) were downloaded from NCBI and used for comparisons.

### Quantitative PCR

The repeat unit copy number was estimated through real time PCR. The primers H2Aq1 (TTTCGGCAAT AGGACTGCTT) and H2Aq2 (ATTGGAATTGGCTGG TAACG) were designed to amplify a histone cluster fragment and RaCHAP-Q1 (GCTTAACAAATGAATCA GTC) and RaCHAP-Q2 (ACTCATTAAACAAAAG GTCA) to amplify a T-complex Chaperonin 5 fragment (Rezende-Teixeira, personal communication), a gene present in single copy in *Drosophila*. The SYBR Green reactions were assembled as instructed by the kit manufacturer (ABgene), with template DNA extracted from testis of adult flies according to Rezende-Teixeira *et al*. (2012). Amplification rates were estimated considering Chaperonin as a single copy gene also in *Rhynchosciara*, using the mathematical method described by Pfaffl (2001).

RNA extractions from salivary glands were performed as described in Rezende-Teixeira *et al*. (2009), and absolute quantitative RT-PCR assays were performed as described by the kit manufacturer (AgPath-ID One-Step RT-PCR, Life Technologies)

The primer set used was: RaH1q1 CGTCCGGTT CATTCAAACTT; RaH1q2 TCACTGAGCCAGCCTTC TTT; RaH3q1 AATTCGTCGCTACCAAAAGAG; RaH3q2 CAGAGCCATAACCGCTGAACT; RaH4q1 ATTACGAAACCAGCCATTCG; RaH4q2 ACCTCCGA AACCGTACAATG; RaH2Aq1 TTTCGGCAATAGGA CTGCTT; RaH2Aq2 ATTGGAATTGGCTGGTAACG; RaH2Bq1 AGAAACGCAAGCGTAAGGAA; RaH2Bq2 TGGTGATGGTCGATCGTTTA.

The experiments were performed in triplicate and dilutions of the cloned histone cluster itself served to establish the reference curve (all the 5.1 kb cloned in pBluescript KS, Stratagene). The primers listed above were designed to produce amplification products containing amplicons of 100-200 bp, all of which were specific products, which was especially important in the case of histone RaH3, which shares many similarities with RaH3.3 (Siviero *et al*., 2006). The amplification efficiencies were determined for all primer pairs (RaH1: 100%; RaH3: 99%; RaH4: 99%; RaH2A: 100%; RaH2B: 99%; RaCHAP: 99%).

### *In situ* hybridization

Salivary gland chromosomes fixed with ethanol-acetic acid (3:1) were squashed in 45% acetic acid. The coverslips were removed by freezing the slides on dry ice. The preparations were then washed in PBS, denatured for 3-5 minutes in NaOH (0.07 M), washed in a series of alcohol and dried. Probes were labelled using the dUTP-digoxigenin Random-primer kit (Roche) according to manufacturer instructions and denatured by heating just before the hybridisation step. The hybridized probes were revealed by fluorescein labelled anti-digoxigenin serum (Roche) and the chromosomes were counterstained with propidium iodide. The preparations were analysed in a laser scanning confocal microscope, LSM-510 (Zeiss), and were considered positive the regions labelled in most of the chromosome optical sections.

## Results and Discussion

### General sequencing results

**Figure 1:**
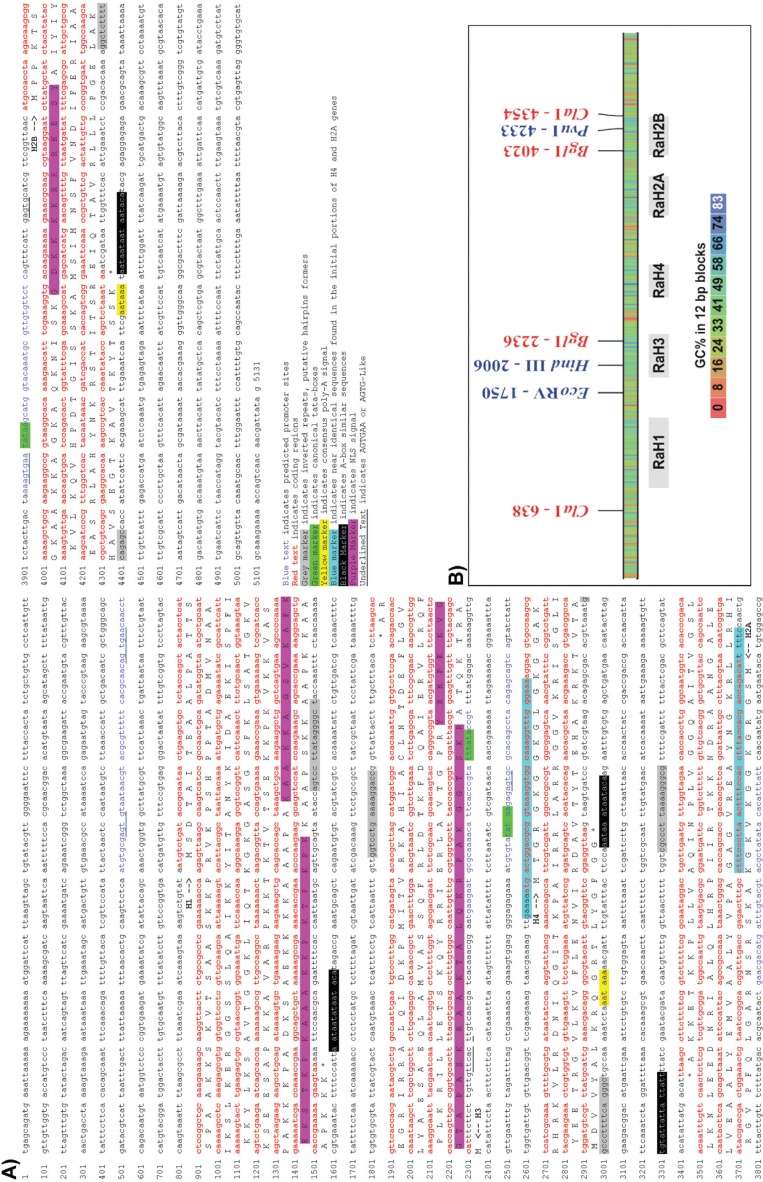
The *Rhynchosciara americana* histone genes. A) Repeat unit sequence, highlighted are putative control elements. B) Restriction map and GC content.

**Figure 2:**
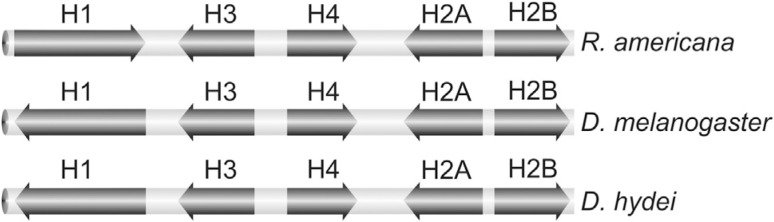
Orientation of histone genes in clusters from different organisms.

A large insert of the histone gene cluster repeat was sub-cloned from a previously isolated recombinant phage and sequenced by shotgun technique. The histone repeat unit in *R. americana* has 5,131 bp (GenBank accession number: AF378198), containing single sites for different restriction enzymes: for *Eco*RV in the RaH1-RaH3 spacer; for *Hind*III in the RaH3 coding region, and for *Pvu*I in the RaH2B portion (Figures 1 and 2). As commonly observed in other histone clusters, the intergenic spacers are AT-rich. The orientation of the individual histone genes is similar to those in *Drosophila* *melanogaster* and *Drosophila hydei*, except for the H1 gene (Figures 1 and 2). The *R. americana* cluster also shares other similarities with its *D. hydei* homologue: like its length, which is only 22 bp shorter, a CG content, 37% for *R. americana* and 38% for *D. hydei* (Table 1), and a strong nucleotide identity in the coding regions (Table 2).

**Table 1:** Nucleotide frequencies of histone repeat unit of *R. americana*, *D. hydei*, *D. melanogaster* (L unit) and *C. thummi*.

Nucleotide	*R. americana*	*D. hydei*	*D. melanogaster*	*C. thummi*
	(5131bp)	(5153bp)	(5041bp)	(6271bp)
Adenine (A)	32.7%	30.3%	28.4%	35.4%
Cytosine (C)	19.2%	18.9%	20.2%	15.6%
Guanine (G)	19.5%	19.1%	20.1%	16.7%
Thymine (T)	28.6%	31.7%	31.3%	32.3%

**Table 2:** Nucleotide identities between *R. americana* and other Diptera histone coding regions and comparison among histone proteins from these organisms, denoting identity and similarity. (Dm – *D. melanogaster*, Dh – *D. hydei*, Ct – *C. thummi*)

	RaH1 x H1	RaH2A x H2A	RaH2B x H2B	RaH3 x H3	RaH4 x H4
Dm (Nucleotide)	49%	79%	80%	80%	78%
Dh (Nucleotide)	48%	82%	80%	84%	82%
Ct (Nucleotide)	58%	82%	79%	83%	85%
Dm (Protein)	45% (63%)	99% (100%)	98% (99%)	99% (100%)	100%
Dh (Protein)	46% (65%)	100%	98% (99%)	100%	100%
Ct (Protein)	38% (54%)	97% (99%)	93% (96%)	100%	100%

Through quantitative real time PCR it was possible to obtain an estimate of 159 copies of the histone cluster unit (± 24 Median Absolute Deviation) in the haploid genome of *R. americana*, which is similar with observations for the genus *Drosophila* (110-150 copies).

### 5' UTR elements

Among the predicted promoter regions, canonical TATA-boxes were found only upstream of the RaH3, RaH4 and RaH2B genes, while RaH1 and RaH2A presumptive promoters have TATA-like sequences. In the putative leader sequences of the genes RaH1, RaH3, RaH4 and RaH2B, the element AGTGAA (or AGTG-like), was found near the start codon of RaH3 and RaH2B, and in the promoter region of RaH1, RaH4 and RaH2B. As observed in *D. hydei* (Kremer and Hennig, 1990), only RaH3 showed an AGTGA block near the start codon (Figure 1). These AGTG-like elements were suggested to have a specific function related to histone expression in the genus *Drosophila* (Matsuo and Yamazaki, 1989; Matsuo, 2003). This hypothesis is now reinforced by the presence of the same AGTG-like elements in the *R. americana* histone repeat unit. The present data actually suggest that the role of these elements can be extrapolated for the Sciaridae family as a whole.

### 3' UTR elements

**Figure 3:**
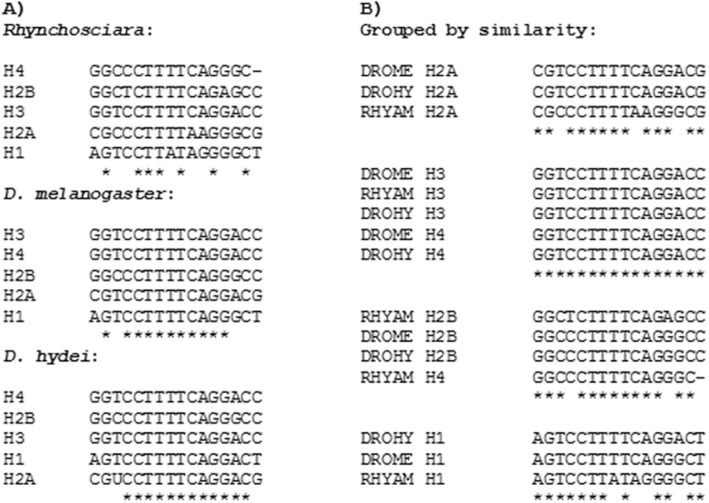
Comparison among hairpins forming inverted repeats from *R. americana*, *D. hydei* and *D. melanogaster*.

In the 3' untranslated region (3' UTR) of each histone gene one palindrome or near-palindrome sequence block was found. These are the hairpin formers (Figures 1 and 3) involved with the regulation of mRNA processing of the cell-cycle dependent histone genes (Birchmeier *et al*., 1982). The inverted repeat present in RaH1 gene is more divergent when compared with the palindromes of the other core histones genes, as was also observed in *D. melanogaster* and *D. hydei* (Kremer and Hennig, 1990). The RaH1 distance from the stop codon (48 bp) is only slightly longer than the one observed in the core histones genes (RaH3: 23 bp; RaH4:40 bp; RaH2A:44 bp; RaH2B: 39bp), and is almost half the distance when compared to the *Drosophila* homologs.

Interestingly, with the exception of RaH4, these inverted repeats found in *R. americana* can be grouped by similarity with their homologs in the *Drosophila* genus (Figure 3), indicating a possible common origin of these elements. The palindromes for H3 histones from *R. americana* and H3/H4 from *D*. *melanogaster* and *D. hydei* are a perfect match. The inverted repeat from RaH4 is imperfect, it is 1 bp shorter in the 5' extremity and similar to the palindromes found in the H2B genes of these three insects. Identity between hairpins of the H4 and H2B genes is also observed in *Chironomus thummi*, which is only a single base different from the correspondent hairpin in *Rhynchosciara*.

Interestingly, the element CAA(T/G)GAGA, which is common in the genus *Drosophila* and is related to the binding of snRNA, or other similar consensus blocks were not found in *R. americana* (Mowry and Steitz, 1987; Soldati and Schumperli, 1988). Further research will thus be needed to determine whether *R. americana* is able to use another sequence to bind snRNAs, or whether it has a mechanism for mRNA maintenance which is somewhat different from the usual one.

Poly-A signals were predicted for the five histone genes, but the prediction algorithm scores were too low for RaH1, RaH3 and RaH2A, so they will not be considered here. The poly-A signals found in RaH4 and RaH2B corresponded to the consensus sequence AATAAA, however no 3' U-rich regions were observed, even putatively downstream CA poly-adenylation sites being present (Manley and Takagaki, 1996). In the annelid *C. variopedatus*, canonical poly-A signals were found for the five genes in the histone cluster (del Gaudio *et al*., 1998). The authors hypothesized that the presence of these termination signals in present in all ancestral histones was substituted during the evolution by the hairpin structure, which corresponds to the observation that these signals are unequally distributed among the histone genes in the *Rhynchosciara* cluster. Double termination signals were reported for the H2B, H3 and H4 genes in *D. melanogaster* by Akhmanova *et al*. (1997). Based on the present data, however, we cannot conclude whether these poly-adenylation signals are actually functional in *R. americana*.

### Other interesting elements

Strikingly, the genes RaH4 and RaH2A share almost the same 44 bases region in the 5' portion (84% identity), starting 5 bp upstream of the start codon (GAAAA) and encompassing the coding segment for the first 13 amino acids, which are, as expected, also very similar (Figure 1A). This identity level is not observed in the same portions of the clusters from *D. melanogaster*, *D. hydei* or *C. thummi*. These 44 bases, including the 5 non-coding base pairs, may reflect the a common origin of these genes, or at least the same origin of this initial domain.

Other noteworthy elements are two identical sequence blocks in the 3' UTR of RaH2A and RaH2B (AATAATAATAATACA), and two similar blocks in the 3' UTR of RaH4 (AAATAAATAATACA) and RaH1 (AATAATATAATACA), all positioned 38-45 bp downstream of the palindromes. These elements resemble A-boxes, and their position suggests that they may play a role in mRNA termination.

### Coding regions

Codon usage determined for the histone genes of *R. americana* (Table S2–S7) shows several points of similarity with the histone codon usage for *D. hydei*. A few differences lay on the *Rhynchosciara* preferential codons: UUG (Leu), CCA and CCG (for Pro), AAA (Lys), CAA (Gln) and GGU (Gly). The only stop codon used in these genes is UAA.

The GC content in the coding regions of the *Rhynchosciara* histone gene cluster ranges from 44.2% (RaH1) to 47.9% (RaH4) with a mean value of 45.9% (Supplementary material, Tables S2–S7). These values are lower than those observed in *D. melanogaster* (mean 51.4%) and *D. hydei* (mean 48.9%). The GC content at the 3^rd^ codon bases (GC3s) is also much lower than that observed in the genus *Drosophila*, 38.4% (average) (Table 3 and Table S1); the lowest GC3s is 34.6% in the RaH3, lower than the GC3s from histone H3 of 16 *Drosophila* species analyzed by Matsuo (2003). In that study the author proposes that a reduction in the population size of a common ancestor of *D. hydei* and *D. americana* may have relaxed the selective pressure, increasing the frequency of substitutions at the 3^rd^ codon bases for A and T. However, the intriguingly low GC3s content in *Rhynchosciara* suggests two possible explanations: 1) the genera *Rhynchosciara* and *Drosophila* would share a common ancestor, so the same conditions of selection pressure and relaxation that occurred for *D. hydei* and *D. americana* could affect *R. americana*; or 2) since *Rhynchosciara* belongs to an ancient branch divergence in Diptera, the common ancestor could already have had a low GC3s content at this locus.

**Table 3:** Bases distribution in the 3^rd^ codon position and GC content of the coding region and in the 3^rd^ codon base.

	T3s	C3s	A3s	G3s	GC3s	GC
H1	0.3893	0.1946	0.4058	0.2412	0.352	0.442
H3	0.4762	0.2381	0.3136	0.1892	0.346	0.473
H2A	0.3980	0.2449	0.3271	0.2376	0.393	0.468
H2B	0.3556	0.3889	0.2913	0.2447	0.483	0.450
H4	0.4048	0.2619	0.3295	0.1951	0.376	0.479
Average of genes	0.4049	0.2567	0.3451	0.2249	0.384	0.459

### Histone proteins

In other organisms, several motifs and domains were identified in histone DNA and protein sequences, such as promoters, nuclear localization signals (NLS) and functional histone domains. In *R. americana*, at least for H1, H3 and H2B, we have identified NLS motifs, and the linking motif of H1 fits all the parameters assumed for others sequences. As expected, all the core characteristic domains for this class of proteins (histone domain/InterPro Search) are present and are nearly identical when compared to *Drosophila* and *Chironomus*.

### Localization of the histone repeat in *Rhynchosciara*

**Figure 4:**
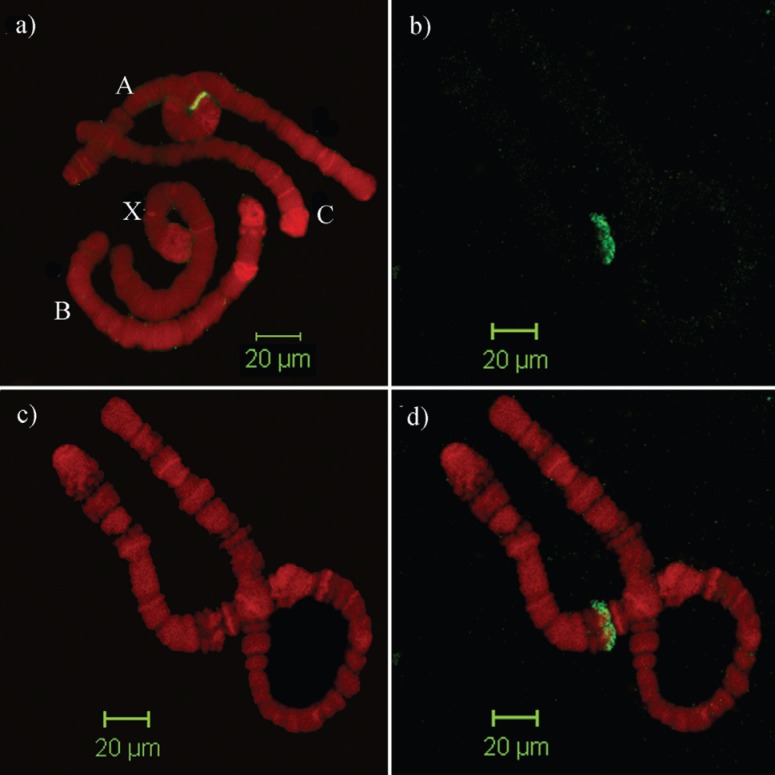
*In situ* hybridization using histone gene repeats as probe labeled with digoxigenin and revealed by a FITC-antidigoxigenin antibody, indicating a single *locus* in region 13 of chromosome A. A) four chromosomes from a single salivary gland cell. B) Split image of chromosome A showing labeled region A13. C) Chromosome counterstained with propidium iodide. D) merged images.

To determine the possible existence of other histone clusters spread across the *R. americana* chromosomes, fluorescent *in situ* hybridization assays were performed. These assays evidenced a single positive band in region 13 of chromosome A when using the repeat unit as probe. The same band was seen under a wide range of stringency conditions (Figure 4). This result suggests the inexistence of other clusters for the replication-dependent histone genes, as is also the case in *D. virilis* (Nagel and Grossbach, 2000) and in *D. americana* (Nagoda *et al*., 2005). This puts in evidence a strong synteny for the histone gene cluster in the genus *Rhynchosciara*, as the same locus was reported to contain the histone cluster in *R. baschanti* and *R. hollaenderi* (Stocker and Gorab, 2000). Southern blot analysis of DNA from salivary glands from a recently discovered species, *Rhynchosciara sp*., showed a unique *Hind*III 5.1 kb band labeling (result not shown) that corresponds to the size of the *R. americana* repeat. The new *Rhynchosciara* species was collected in Ubatuba, SP, and its proper description is in progress (Machado-Santelli G.M., unpublished results).

These results indicate that the unique cluster present in the 13A locus, containing all the replication-dependent histone genes, may be a common feature among *Rhynchosciara* species.

### Transcription profile in salivary glands

To analyze the role of these canonical histones during polytene chromosome development, we determined the transcriptional profile in salivary glands of each of the histone genes present in the cluster of *Rhynchosciara*. The expression levels were measured by absolute quantification of the respective transcripts. We believe that this information can contribute to a better understanding of chromatin alterations occurring during the gene amplification or polytenization processes, and to the understanding the role of histone variants such as RaH3.3 detected in this tissue (Siviero *et al*., 2006).

**Figure 5:**
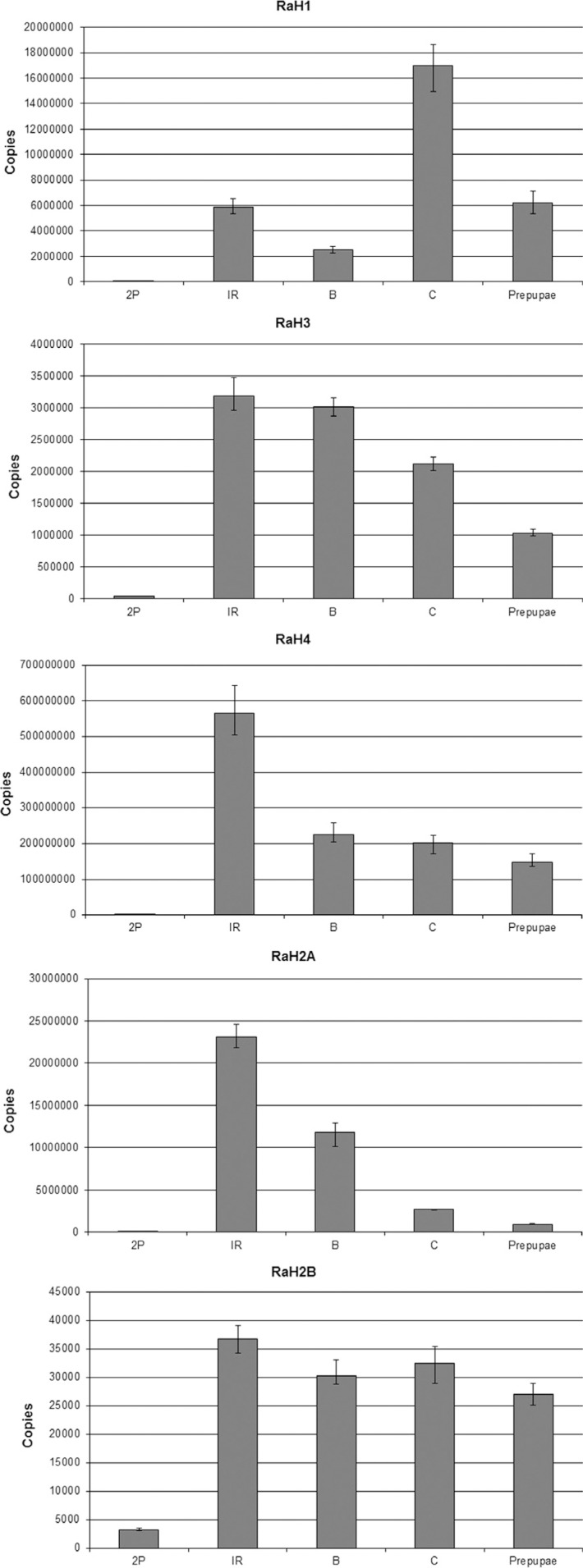
Transcription profiles of the *Rhynchosciara americana* canonical histone genes during larval development through absolute quantification. The vertical axis shows transcript numbers for each developmental period: 2P – second period of the fourth Instar; IR – 3^rd^ period of the fourth instar, corresponding to initial cocoon spinning; B – 4^th^ period of the fourth instar, corresponding to Puff 2B formation; 5^th^ period of fourth instar corresponding to Puff 3C formation; and 6^th^ period of fourth instar corresponding the prepupal stage. Shown are means and standard errors of the mean for the triplicate samples.

The expression profiles (Figure 5) show that all histones have greatly increased transcription levels from the moment on when cocoon spinning starts. This was expected, since this period encompasses the last polytenization cycle and the beginning of gene amplification in certain puffs.

Interestingly, the RaH4 mRNA levels were much higher than those for the other histone genes at all stages analyzed. This may reflect an increased consumption of protein due to a higher turnover, or it may be a consequence of a shorter half-life of this mRNA, since in *Rhynchosciara* this gene has an imperfect hairpin in its 3' UTR portion.

The lowest detected mRNA levels were observed for RaH2A and RaH2B, which may be a direct consequence of the use of other variants of these proteins (Park and Luger, 2008; Lindner, 2008), very common in other organisms, or can be caused by a longer half-life of the mRNA.

While the nucleosomal histones (core histones) presented a transcription peak in the initial cocoon-spinning period (IR), with a consequent decrease in other stages, the Histone RaH1 showed a higher level of transcription in the period of 3C puff expansion. Even in prepupae, the RaH1 levels were higher than during IR. This pattern of transcription may be related to chromatin modifications necessary for gland histolysis and later metamorphosis, such as higher chromosome condensation (Brandão *et al*., 2014).

## Final remarks

The characterization of the *R. americana* histone gene cluster adds new insights to this evolutionarily and genomically poorly studied system. These data indicate that *R. americana* has a single cluster of the five histone genes repeated approximately 159 times. Sequencing and analysis of the histone clusters from the other known *Rhynchosciara* species (*R. baschanti*, *R. hollaenderi*, *R. milleri*, *R. papaveroi* and *R. sp*) will now be greatly facilitated and could introduce important molecular data for an evolutionarily much more ancient dipteran genus than *Drosophila*, possibly providing relevant data on the evolution of the Diptera branch as a whole.

## Supplementary material

The following online material is available for this article:

Table S1 - Nucleotide base distribution in the 3^rd^ codon position and GC content of the coding region and in the 3^rd^ codon base. (codon Bias Index).

Table S2 - Codon usage for *Rhynchosciara americana* Histone H1.

Table S3 - Codon usage for *Rhynchosciara americana* Histone H3.

Table S4 - Codon usage for *Rhynchosciara americana* Histone H2A.

Table S5 - Codon usage for *Rhynchosciara americana* Histone H2B.

Table S6 - Codon usage for *Rhynchosciara americana* Histone H4.

Table S7 - Average codon usage for *Rhynchosciara americana* histone genes.
