# Supplementary material for: The histone genes cluster in Rhynchosciara americana and its transcription profile in salivary glands during larval development
Source: Genet Mol Biol. 2016 Oct 10;39(4):580–8. doi: 10.1590/1678-4685-GMB-2015-0306 (PMC5127150; doi:10.1590/1678-4685-GMB-2015-0306)
Supplement: Supplementary file 1 [file 1415-4757-gmb-1678-4685-GMB-2015-0306-Suppl02.pdf]

Table S2 – Codon usage for *Rhynchosciara americana* Histone H1.

Phe	UUU	0	0.00	Ser	UCU	5	1.30	Tyr	UAU	0	0.00	Cys	UGU	0	0.00
	UUC	2	2.00		UCC	3	0.78		UAC	3	2.00		UGC	0	0.00
Leu	UUA	0	0.00		UCA	5	1.30	TER	UAA	1	3.00	TER	UGA	0	0.00
	UUG	3	3.00		UCG	4	1.04		UAG	0	0.00	Trp	UGG	0	0.00
	CUU	1	1.00	Pro	CCU	2	0.38	His	CAU	1	2.00	Arg	CGU	1	2.00
	CUC	1	1.00		CCC	1	0.19		CAC	0	0.00		CGC	0	0.00
	CUA	1	1.00		CCA	10	1.90	Gln	CAA	2	2.00		CGA	0	0.00
	CUG	0	0.00		CCG	8	1.52		CAG	0	0.00		CGG	0	0.00
Ile	AUU	5	1.88	Thr	ACU	6	1.60	Asn	AAU	2	2.00	Ser	AGU	4	1.04
	AUC	1	0.38		ACC	6	1.60		AAC	0	0.00		AGC	2	0.52
	AUA	2	0.75		ACA	1	0.27	Lys	AAA	35	1.17	Arg	AGA	1	2.00
Met	AUG	2	1.00		ACG	2	0.53		AAG	25	0.83		AGG	1	2.00
Val	GUU	5	2.00	Ala	GCU	17	1.62	Asp	GAU	4	2.00	Gly	GGU	5	2.22
	GUC	1	0.40		GCC	6	0.57		GAC	0	0.00		GGC	3	1.33
	GUA	2	0.80		GCA	18	1.71	Glu	GAA	6	1.50		GGA	1	0.44
	GUG	2	0.80		GCG	1	0.10		GAG	2	0.50		GGG	0	0.00

222 codons in H1 (used Universal Genetic code)
