# Supplementary material for: The histone genes cluster in Rhynchosciara americana and its transcription profile in salivary glands during larval development
Source: Genet Mol Biol. 2016 Oct 10;39(4):580–8. doi: 10.1590/1678-4685-GMB-2015-0306 (PMC5127150; doi:10.1590/1678-4685-GMB-2015-0306)
Supplement: Supplementary file 2 [file 1415-4757-gmb-1678-4685-GMB-2015-0306-Suppl07.pdf]

Table S7 – Average codon usage for *Rhynchosciara americana* histone genes.

Phe	UUU	2	0.36	Ser	UCU	9	1.15	Tyr	UAU	7	0.78	Cys	UGU	1	2.00
	UUC	9	1.64		UCC	5	0.64		UAC	11	1.22		UGC	0	0.00
Leu	UUA	5	0.64		UCA	7	0.89	TER	UAA	5	3.00	TER	UGA	0	0.00
	UUG	32	4.09		UCG	9	1.15		UAG	0	0.00	Trp	UGG	0	0.00
	CUU	2	0.26	Pro	CCU	5	0.54	His	CAU	5	1.00	Arg	CGU	28	3.17
	CUC	1	0.13		CCC	2	0.22		CAC	5	1.00		CGC	10	1.33
	CUA	4	0.51		CCA	19	2.05	Gln	CAA	17	1.79		CGA	8	0.91
	CUG	3	0.38		CCG	11	1.19		CAG	2	0.21		CGG	3	0.34
Ile	AUU	22	1.83	Thr	ACU	13	1.18	Asn	AAU	8	1.00	Ser	AGU	8	1.02
	AUC	11	0.92		ACC	19	1.73		AAC	8	1.00		AGC	9	1.15
	AUA	3	0.25		ACA	8	0.73	Lys	AAA	68	1.15	Arg	AGA	3	0.34
Met	AUG	12	1.00		ACG	4	0.36		AAG	50	0.85		AGG	1	0.11
Val	GUU	20	2.00	Ala	GCU	39	1.61	Asp	GAU	10	1.25	Gly	GGU	34	2.57
	GUC	9	0.90		GCC	23	0.95		GAC	6	0.75		GGC	7	0.53
	GUA	4	0.40		GCA	30	1.24	Glu	GAA	28	1.75		GGA	11	0.83
	GUG	7	0.70		GCG	5	0.21		GAG	4	0.25		GGG	1	0.08

712 codons in Average of genes (used Universal Genetic code)
