# Supplementary material for: The histone genes cluster in Rhynchosciara americana and its transcription profile in salivary glands during larval development
Source: Genet Mol Biol. 2016 Oct 10;39(4):580–8. doi: 10.1590/1678-4685-GMB-2015-0306 (PMC5127150; doi:10.1590/1678-4685-GMB-2015-0306)
Supplement: Supplementary file 3 [file 1415-4757-gmb-1678-4685-GMB-2015-0306-Suppl01.pdf]

Table S1 – Nucleotide base distribution in the 3rd codon position and GC content of the coding region and in the 3rd codon base (codon Bias Index).

	**T3s**	**C3s**	**A3s**	**G3s**	**CAI**	**CBI**	**Fop**	**Nc**	**GC3s**	**GC**	**L_sym**	**L_aa**
**H1**	0.3893	0.1946	0.4058	0.2412	0.364	0.092	0.498	47.79	0.352	0.442	219	221
**H3**	0.4762	0.2381	0.3136	0.1892	0.330	0.240	0.556	35.81	0.346	0.473	133	136
**H2A**	0.3980	0.2449	0.3271	0.2376	0.270	0.184	0.516	35.51	0.393	0.468	122	124
**H2B**	0.3556	0.3889	0.2913	0.2447	0.275	0.122	0.508	48.83	0.483	0.450	120	123
**H4**	0.4048	0.2619	0.3295	0.1951	0.254	0.186	0.525	37.76	0.376	0.479	101	103
**Average of genes**	0.4049	0.2567	0.3451	0.2249	0.307	0.157	0.518	43.24	0.384	0.459	695	707

CAI (codon adaptation index), CBI (codon Bias Index), Fop (frequency of optimal codons), Nc (effective number of codons), L sym (synonymous codons), L aa (total of translatable codons).
