# Supplementary material for: The histone genes cluster in Rhynchosciara americana and its transcription profile in salivary glands during larval development
Source: Genet Mol Biol. 2016 Oct 10;39(4):580–8. doi: 10.1590/1678-4685-GMB-2015-0306 (PMC5127150; doi:10.1590/1678-4685-GMB-2015-0306)
Supplement: Table S3 [file 1415-4757-gmb-1678-4685-GMB-2015-0306-Suppl03.pdf]

Table S3 – Codon usage for *Rhynchosciara americana* Histone H3.

Phe	UUU	0	0.00	Ser	UCU	0	0.00	Tyr	UAU	1	0.67	Cys	UGU	1	2.00
	UUC	4	2.00		UCC	0	0.00		UAC	2	1.33		UGC	0	0.00
Leu	UUA	1	0.50		UCA	1	1.00	TER	UAA	1	3.00	TER	UGA	0	0.00
	UUG	8	4.00		UCG	1	1.00		UAG	0	0.00	Trp	UGG	0	0.00
	CUU	1	0.50	Pro	CCU	2	1.33	His	CAU	2	2.00	Arg	CGU	11	3.67
	CUC	0	0.00		CCC	0	0.00		CAC	0	0.00		CGC	4	1.33
	CUA	0	0.00		CCA	3	2.00	Gln	CAA	8	2.00		CGA	1	0.33
	CUG	2	1.00		CCG	1	0.67		CAG	0	0.00		CGG	1	0.33
Ile	AUU	4	1.71	Thr	ACU	4	1.60	Asn	AAU	1	2.00	Ser	AGU	2	2.00
	AUC	3	1.29		ACC	4	1.60		AAC	0	0.00		AGC	2	2.00
	AUA	0	0.00		ACA	2	0.80	Lys	AAA	7	1.08	Arg	AGA	1	0.33
Met	AUG	3	1.00		ACG	0	0.00		AAG	6	0.92		AGG	0	0.00
Val	GUU	6	4.00	Ala	GCU	7	1.56	Asp	GAU	2	1.00	Gly	GGU	6	3.43
	GUC	0	0.00		GCC	4	0.89		GAC	2	1.00		GGC	0	0.00
	GUA	0	0.00		GCA	5	1.11	Glu	GAA	7	2.00		GGA	1	0.57
	GUG	0	0.00		GCG	2	0.44		GAG	0	0.00		GGG	0	0.00

137 codons in H3 (used Universal Genetic code)
