# Supplementary material for: The histone genes cluster in Rhynchosciara americana and its transcription profile in salivary glands during larval development
Source: Genet Mol Biol. 2016 Oct 10;39(4):580–8. doi: 10.1590/1678-4685-GMB-2015-0306 (PMC5127150; doi:10.1590/1678-4685-GMB-2015-0306)
Supplement: Table S4 [file 1415-4757-gmb-1678-4685-GMB-2015-0306-Suppl04.pdf]

Table S4 – Codon usage for *Rhynchosciara americana* Histone H2A.

Phe	UUU	1	2.00	Ser	UCU	2	3.00	Tyr	UAU	3	2.00	Cys	UGU	0	0.00
	UUC	0	0.00		UCC	0	0.00		UAC	0	0.00		UGC	0	0.00
Leu	UUA	4	1.60		UCA	1	1.50	TER	UAA	1	3.00	TER	UGA	0	0.00
	UUG	9	3.60		UCG	1	1.50		UAG	0	0.00	Trp	UGG	0	0.00
	CUU	0	0.00	Pro	CCU	0	0.00	His	CAU	0	0.00	Arg	CGU	7	3.82
	CUC	0	0.00		CCC	0	0.00		CAC	2	2.00		CGC	1	0.55
	CUA	2	0.80		CCA	3	2.40	Gln	CAA	3	1.50		CGA	1	0.55
	CUG	0	0.00		CCG	2	1.60		CAG	1	0.50		CGG	1	0.55
Ile	AUU	4	2.00	Thr	ACU	0	0.00	Asn	AAU	1	0.29	Ser	AGU	0	0.00
	AUC	2	1.00		ACC	3	4.00		AAC	6	1.71		AGC	0	0.00
	AUA	0	0.00		ACA	0	0.00	Lys	AAA	6	0.92	Arg	AGA	1	0.55
Met	AUG	2	1.00		ACG	0	0.00		AAG	7	1.08		AGG	0	0.00
Val	GUU	3	1.33	Ala	GCU	8	2.00	Asp	GAU	0	0.00	Gly	GGU	10	2.86
	GUC	3	1.33		GCC	4	1.00		GAC	2	2.00		GGC	1	0.29
	GUA	2	0.89		GCA	3	0.75	Glu	GAA	6	1.71		GGA	3	0.86
	GUG	1	0.44		GCG	1	0.25		GAG	1	0.29		GGG	0	0.00

125 codons in H2A (used Universal Genetic code)
