# Supplementary material for: The histone genes cluster in Rhynchosciara americana and its transcription profile in salivary glands during larval development
Source: Genet Mol Biol. 2016 Oct 10;39(4):580–8. doi: 10.1590/1678-4685-GMB-2015-0306 (PMC5127150; doi:10.1590/1678-4685-GMB-2015-0306)
Supplement: Table S5 [file 1415-4757-gmb-1678-4685-GMB-2015-0306-Suppl05.pdf]

Table S5 – Codon usage for *Rhynchosciara americana* Histone H2B.

Phe	UUU	1	1.00	Ser	UCU	2	0.92	Tyr	UAU	2	0.80	Cys	UGU	0	0.00
	UUC	1	1.00		UCC	1	0.46		UAC	3	1.20		UGC	0	0.00
Leu	UUA	0	0.00		UCA	0	0.00	TER	UAA	1	3.00	TER	UGA	0	0.00
	UUG	5	5.00		UCG	3	1.38		UAG	0	0.00	Trp	UGG	0	0.00
	CUU	0	0.00	Pro	CCU	1	1.00	His	CAU	1	0.67	Arg	CGU	2	1.71
	CUC	0	0.00		CCC	1	1.00		CAC	2	1.33		CGC	2	1.71
	CUA	1	1.00		CCA	2	2.00	Gln	CAA	3	2.00		CGA	2	1.71
	CUG	0	0.00		CCG	0	0.00		CAG	0	0.00		CGG	1	0.86
Ile	AUU	5	1.67	Thr	ACU	1	0.50	Asn	AAU	2	1.00	Ser	AGU	2	0.92
	AUC	3	1.00		ACC	5	2.50		AAC	2	1.00		AGC	5	2.31
	AUA	1	0.33		ACA	2	1.00	Lys	AAA	11	1.05	Arg	AGA	0	0.00
Met	AUG	3	1.00		ACG	0	0.00		AAG	10	0.95		AGG	0	0.00
Val	GUU	2	1.33	Ala	GCU	6	1.71	Asp	GAU	1	0.67	Gly	GGU	4	2.67
	GUC	2	1.33		GCC	5	1.43		GAC	2	1.33		GGC	1	0.67
	GUA	0	0.00		GCA	2	0.57	Glu	GAA	5	1.67		GGA	1	0.67
	GUG	2	1.33		GCG	1	0.29		GAG	1	0.33		GGG	0	0.00

124 codons in H2B (used Universal Genetic code)
