# Supplementary material for: The histone genes cluster in Rhynchosciara americana and its transcription profile in salivary glands during larval development
Source: Genet Mol Biol. 2016 Oct 10;39(4):580–8. doi: 10.1590/1678-4685-GMB-2015-0306 (PMC5127150; doi:10.1590/1678-4685-GMB-2015-0306)
Supplement: Table S6 [file 1415-4757-gmb-1678-4685-GMB-2015-0306-Suppl06.pdf]

Table S6 – Codon usage for *Rhynchosciara americana* Histone H4.

Phe	UUU	0	0.00	Ser	UCU	0	0.00	Tyr	UAU	1	0.50	Cys	UGU	0	0.00
	UUC	2	2.00		UCC	1	6.00		UAC	3	1.50		UGC	0	0.00
Leu	UUA	0	0.00		UCA	0	0.00	TER	UAA	1	3.00	TER	UGA	0	0.00
	UUG	7	5.20		UCG	0	0.00		UAG	0	0.00	Trp	UGG	0	0.00
	CUU	0	0.00	Pro	CCU	0	0.00	His	CAU	1	1.00	Arg	CGU	7	3.00
	CUC	0	0.00		CCC	0	0.00		CAC	1	1.00		CGC	3	1.29
	CUA	0	0.00		CCA	1	4.00	Gln	CAA	1	1.00		CGA	4	1.71
	CUG	1	0.75		CCG	0	0.00		CAG	1	1.00		CGG	0	0.00
Ile	AUU	4	2.00	Thr	ACU	2	1.00	Asn	AAU	2	2.00	Ser	AGU	0	0.00
	AUC	2	1.00		ACC	1	0.50		AAC	0	0.00		AGC	0	0.00
	AUA	0	0.00		ACA	3	1.50	Lys	AAA	9	1.64	Arg	AGA	0	0.00
Met	AUG	2	1.00		ACG	2	1.00		AAG	2	0.36		AGG	0	0.00
Val	GUU	4	1.78	Ala	GCU	1	0.57	Asp	GAU	3	2.00	Gly	GGU	9	2.12
	GUC	3	1.33		GCC	4	2.29		GAC	0	0.00		GGC	2	0.47
	GUA	0	0.00		GCA	2	1.14	Glu	GAA	4	2.00		GGA	5	1.18
	GUG	2	0.89		GCG	0	0.00		GAG	0	0.00		GGG	1	0.24

104 codons in H4 (used Universal Genetic code)
